# The Expression of Vitamin D Receptor on Peripheral Blood Mononuclear Cells in Patients with Psoriasis

**DOI:** 10.3390/ijms251910677

**Published:** 2024-10-03

**Authors:** Azin Jasmin Zanghaneh, Andrea Elmelid, Martin Gillstedt, Omar Ahmic, Bengt Andersson, Amra Osmancevic

**Affiliations:** 1 Department of Dermatology and Venereology, Västmanlands Sjukhus Västerås, 721 89 Västerås, Sweden; 2 Department of Dermatology, Institute of Clinical Sciences, Sahlgrenska Academy, University of Gothenburg, 413 45 Gothenburg, Sweden; andrea.elmelid@regiondalarna.se (A.E.); martin.gillstedt@vgregion.se (M.G.); amra.osmancevic@vgregion.se (A.O.); 3 Center for Clinical Research, Uppsala University, 791 82 Falun, Sweden; 4 Department of Dermatology and Venereology, Sahlgrenska University Hospital, Region Västra Götaland, 413 45 Gothenburg, Sweden; 5 Accident and Emergency Department, Northern Älvsborg County Hospital, 461 73 Trollhättan, Sweden; omar.ahmic@vgregion.se; 6 Department of Clinical Immunology and Transfusion Medicine, Sahlgrenska University Hospital, Region Västra Götaland, 413 45 Gothenburg, Sweden; bengt.a.andersson@vgregion.se

**Keywords:** Psoriasis, tumor necrosis factor inhibitor, vitamin D, vitamin D receptor, CD3^+^ lymphocytes, CD14^+^ monocytes

## Abstract

Vitamin D plays an important role in psoriasis, but its involvement in pathogenesis remains unclear. This study aimed to evaluate vitamin D receptor (VDR) expression on peripheral blood mononuclear cells (PBMCs) in patients with psoriasis and healthy controls and to study the effects of the Etanercept treatment on VDR expression on PBMCs in patients with psoriasis. Twenty patients with moderate to severe psoriasis received treatment with Etanercept for 24 weeks. The age- and sex-matched controls did not receive any intervention. VDR expression on CD3^+^ lymphocytes and CD14^+^ monocytes, and serum levels of total and free 25-hydroxyvitamin D (25(OH)D) and 1,25-dihydroxy vitamin D (1,25(OH)_2_D) were analyzed at baseline, after 10–12 weeks, and after 24 weeks in both groups. VDR expression was analyzed using flow cytometry. We observed higher expression of the VDR on CD14^+^ monocytes in psoriasis patients compared to healthy controls at baseline. This difference was no longer significant after 24 weeks of the Etanercept treatment. The patients with psoriasis improved clinically. However, VDR expression was unaltered during the Etanercept treatment, and there was no correlation between VDR expression and disease severity.

## 1. Introduction

Psoriasis is a chronic, immune-mediated disease affecting approximately 125 million people worldwide. Psoriasis is associated with various comorbidities, such as cardiometabolic disease, psoriatic arthritis, and depression [1]. The most common type of psoriasis is plaque psoriasis, accounting for approximately 80–90% of cases [2]. The complex pathogenesis of psoriasis is not completely understood, but convincing evidence suggests that it is a systemic immune-mediated disease in which T cells have a central role in the activation and production of inflammatory cytokines [3]. Activated myeloid dendritic cells secrete interleukin 23 (IL-23), which plays a central role in both supporting the survival and proliferation of T helper 17 (Th17) cells and T helper 22 (Th22) cells [1]. Th17 secretes tumor necrosis factor α (TNF-α), IL-17, and IL-22. The IL-23-mediated activation of the Th17 pathway is thought to be predominant in the pathogenesis of plaque psoriasis [1]. Vitamin D deficiency has historically been associated with adult osteomalacia and childhood rickets. However, our knowledge regarding vitamin D has now expanded, and vitamin D deficiency has been linked to chronic systemic illnesses such as multiple sclerosis, cancer, autoimmune diseases, cardiovascular diseases, and type 1 diabetes [3]. Tumor necrosis factor α inhibitors (TNFi) are a well-established treatment for moderate to severe psoriasis [4]. These drugs inhibit TNF-α, thereby diminishing the downstream inflammatory cascade which plays a key role in the pathogenesis of psoriasis [1]. 1,25-dihydroxyvitamin D (1,25(OH)_2_D), along with its analogues, may suppress the expression of TNF-α and Th1-related chemokines, suggesting a potential anti-TNF-α effect [5]. It remains uncertain whether vitamin D is directly involved in the development of psoriasis. However, it is established that vitamin D plays a significant role in the disease, and vitamin D analogues are effectively used as topical treatments for plaque psoriasis [2,3]. The cellular actions of 1,25(OH)_2_D, the active form of vitamin D, are mediated by the vitamin D receptor (VDR) [6]. The VDR is a member of the superfamily of the nuclear steroid hormone receptors and is activated by the binding of 1,25(OH)_2_D [6,7]. Following ligand binding, the formation of two independent protein interaction surfaces on the receptor are formed: one that is required for the recruitment of co-regulators necessary for gene modulation and one that forms a heterodimer with the retinoid X receptor and regulates the expression of genes via binding to vitamin D-responsive elements [6,7]. VDR expression has been discovered in almost every tissue, including immune cells, in both monocytes and in activated T and B lymphocytes. This suggests that 1,25(OH)_2_D has an immunoregulatory function [8,9,10]. Recent studies have highlighted the significance of 1,25(OH)_2_D–VDR signaling and its impact on immune function as a link between 1,25(OH)_2_D and the development of inflammatory diseases [6]. T cells play a central role in both the development of inflammatory diseases and in protective immunity. A variety of studies have found that the expression and activity of the VDR are of great importance in the development, differentiation, and effector function of T cells [6,8]. Vitamin D supports the differentiation of naïve T cell into regulatory T cells, enhancing the production of anti-inflammatory cytokines while suppressing the production of pro-inflammatory cytokines, such as IL-17A [2].

The primary aim of this study was to examine if VDR expression on peripheral blood mononuclear cells (PBMCs) differs between patients with psoriasis and healthy controls. Furthermore, the study aimed to investigate the impact of Etanercept treatment on the VDR expression on PBMCs. 

## 2. Results

### 2.1. Demographics

Demographic data, including concomitant medication and possible confounding factors for vitamin D status, are presented in Table 1 [5]. In the psoriasis group, seventeen patients completed the study, and three dropouts occurred: one patient due to a side-effect, one due to lack of compliance, and one due to lack of treatment response. One dropout occurred among the healthy controls due to personal reasons. For three participants among the healthy controls the VDR data was not available at each visit.

### 2.2. Comparisons between Patients with Psoriasis and Healthy Controls at Baseline and after 24 Weeks Regarding VDR expression on CD3^+^ Lymphocytes and CD14^+^ Monocytes

There was no significant difference in VDR expression on CD3^+^ lymphocytes in patients with psoriasis compared to healthy controls at baseline (*p* = 0.83). VDR expression on CD14^+^ monocytes was higher in patients with psoriasis compared to healthy controls at baseline (*p* = 0.015) (Table 2). When comparing patients with psoriasis to healthy controls at week 24, there were no statistically significant differences in VDR expression on CD3^+^ lymphocytes (*p* = 0.75) or CD14^+^ monocytes (*p* = 0.56).

### 2.3. Changes during the 24-Week Follow-Up in VDR Expression on CD3^+^ Lymphocytes and CD14^+^ Monocytes in Patients with Psoriasis and Healthy Controls

**Figure 1:**
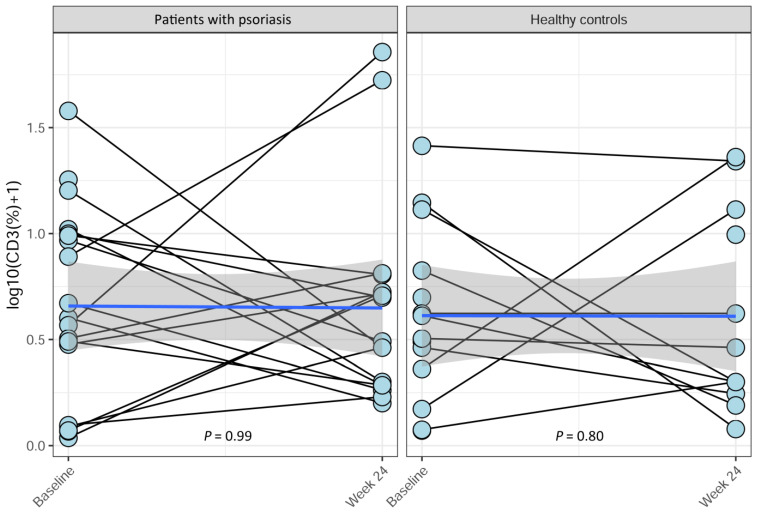
Changes in VDR expression on CD3^+^ lymphocytes over time in patients with psoriasis and in healthy controls. Psoriasis patients initiated the Etanercept treatment at baseline and continued through to week 24. Healthy controls received no treatment. There was no significant change in VDR expression on CD3^+^ cells in patients with psoriasis or in healthy controls over the 24-week period (*p* = 0.99, and *p* = 0.80). The *p*-values were obtained using Spearman’s correlation test, stratified with respect to patient. The blue lines are fitted linear regression lines, and the gray areas are 95% confidence intervals for the regression lines.

**Figure 2:**
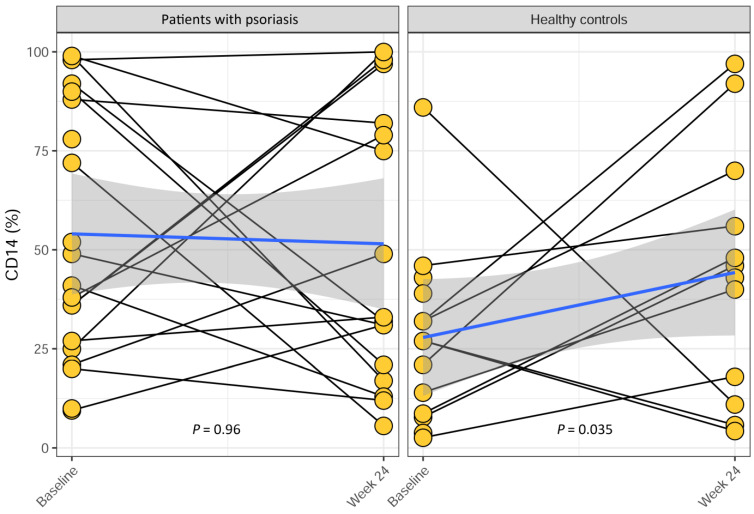
Changes in VDR expression on CD14^+^ monocytes over time in patients with psoriasis and in healthy controls. Psoriasis patients initiated the Etanercept treatment at baseline and continued through to week 24. Healthy controls received no treatment. There was no significant change in VDR expression on CD14^+^ cells in patients with psoriasis over the 24-week period (*p* = 0.96), while the expression increased in healthy controls (*p* = 0.035). The *p*-values were obtained using Spearman’s correlation test, stratified with respect to patient. The blue lines are fitted linear regression lines, and the gray areas are 95% confidence intervals for the regression lines.

Treatment with Etanercept did not alter VDR expression on CD3^+^ and CD14^+^ cells in patients with psoriasis over the 24-week period (*p* = 0.99, *p* = 0.96) (Figure 1 and Figure 2). Healthy controls, who received no treatment, had no change in VDR expression on CD3^+^ cells over the 24-week period (*p* = 0.80) (Figure 1), but VDR expression on CD14^+^ cells increased over time (*p* = 0.035) (Figure 2).

### 2.4. Serum Vitamin D Levels at Baseline and at Week 24 in Patients with Psoriasis and Healthy Controls

Higher total and free 25-hydroxyvitamin D (25(OH)D) levels were observed in patients with psoriasis compared to healthy controls at baseline (*p* = 0.018 and *p* = 0.001) (Table 2) [5]. At the 24-week follow-up, total and free 25(OH)D and 1,25(OH)_2_D remained unaltered in the psoriasis group, while both total and free 25(OH)D increased in the healthy controls (Table 2) [5].

### 2.5. Correlations between VDR Expression on CD3^+^ and CD14^+^ Cells in Patients with Psoriasis and Healthy Controls

**Figure 3:**
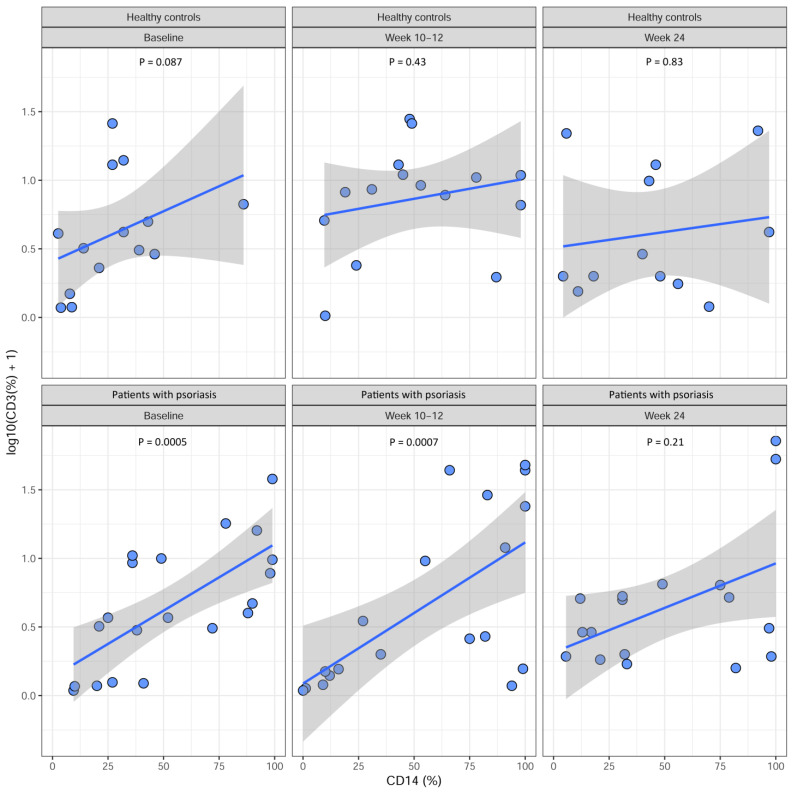
Correlations between VDR expression on CD3^+^ lymphocytes and CD14^+^ monocytes at baseline, after 10–12 weeks, and after 24 weeks. Psoriasis patients initiated the Etanercept treatment at baseline and continued through to week 24, while healthy controls received no treatment. The *p*-values were obtained using Spearman’s correlation test. The blue lines are fitted linear regression lines, and the gray areas are 95% confidence intervals for the regression lines.

There was a significant positive correlation between VDR expression on CD3^+^ lymphocytes and CD14^+^ monocytes in patients with psoriasis at baseline (*p* = 0.0005). This correlation remained after 10–12 weeks of treatment (*p* = 0.0007), but no significant correlation was found after 24 weeks of treatment (*p* = 0.21) (Figure 3). Likewise, no correlation was found in healthy controls (Figure 3).

### 2.6. Correlations between VDR Expression on CD3^+^ and CD14^+^ Cells, and Serum Levels of Vitamin D Metabolites

There was no correlation between baseline serum levels of total 25(OH)D and 1,25(OH)_2_D and baseline VDR expression on CD3^+^ and CD14^+^ cells. There were no correlations between baseline serum levels of total 25(OH)D, free 25(OH)D, or 1,25(OH)_2_D and changes in VDR expression on CD3^+^ and CD14^+^ cells in patients with psoriasis or in healthy controls. Furthermore, no correlations were found between the changes in total 25(OH)D, free 25(OH)D, or 1,25(OH) levels and the changes in VDR expression on CD3^+^ and CD14^+^ cells in either group.

When looking at the entire study population of both patients with psoriasis and healthy controls, there were no correlations between baseline serum levels of total 25(OH)D and free 25(OH)D and baseline VDR expression on CD3^+^ and CD14^+^ cells. Furthermore, no correlations were found between changes in serum levels of total and free 25(OH)D and changes in VDR expression on CD3^+^ and CD14^+^ cells.

### 2.7. Correlations between VDR Expression on CD3^+^ and CD14^+^ Cells, and Clinical Outcomes

Treatment with Etanercept effectively reduced the PASI score in patients with psoriasis (Table 2) [5]. No correlations were found between the PASI score at baseline and VDR expression on CD3^+^ and CD14+ cells at baseline. No significant correlations were found between the change in VDR expression on CD3^+^ and CD14^+^ cells and the change in PASI score over time. Additionally, there were no correlations between baseline PASI scores and changes in VDR expression on CD3^+^ and CD14^+^ cells.

## 3. Discussion

To the best of our knowledge, this is the first report describing VDR expression on CD3^+^ lymphocytes and CD14^+^ monocytes in patients with psoriasis.

In this prospective observational study, we observed a significant difference in VDR expression on CD14^+^ monocytes when comparing patients with psoriasis and healthy controls at baseline, with a higher expression in the psoriasis group. The Etanercept treatment improved the disease but did not alter VDR expression on CD3^+^ lymphocytes and CD14^+^ monocytes in patients with psoriasis. However, healthy controls showed a significant increase in VDR expression on CD14^+^ monocytes during the study. This may be attributed to the increase in both total and free 25(OH)D levels observed in healthy controls, whereas vitamin D levels remained stable in patients with psoriasis. The cause of the increase in 25(OH)D levels in healthy controls is unclear, but may be due to differences in sun behavior between the groups [5]. Although our data did not establish a significant correlation between serum 25(OH)D levels and VDR expression on PBMCs, this observation raises the possibility of a concurrent upregulation in VDR expression among healthy controls. It also suggests that total and free 25(OH)D levels may regulate VDR expression in the analyzed immune cells. This aligns with a previous randomized controlled trial (RCT) involving dialysis patients, which reported that patients treated with 50,000 IU of cholecalciferol twice a week showed an increase in serum 25(OH)D concentration and monocyte VDR expression. In contrast, the control group exhibited no change in serum 25(OH)D levels and a decrease in VDR expression [11]. In another RCT involving monozygotic twins, an intervention with cholecalciferol supplementation (2000 IU/day) was performed for 2 months. The findings demonstrated both an increase in serum 25(OH)D levels and in VDR gene expression. No significant change in serum 25(OH)D levels were seen in the control group [12]. In contrast, a RCT including pre-frail older adults analyzing the effect of cholecalciferol supplementation on VDR expression in monocytes found that the group receiving cholecalciferol supplementation (4000 IU/day) showed a significantly higher increase in 25(OH)D levels compared to the placebo group, but this did not change VDR expression in monocytes. Additionally, no significant difference between the groups was found regarding the change from baseline VDR expression [13].

In the present study, the difference regarding higher VDR expression on CD14^+^ cells found at baseline in patients with psoriasis compared to healthy controls was no longer observed after 24 weeks of treatment with Etanercept, indicating that the levels of vitamin D more likely affected VDR expression than the anti-inflammatory effects of TNFi treatment in psoriasis.

We found a positive correlation between VDR expression on CD3^+^ lymphocytes and CD14^+^ monocytes in patients with psoriasis at baseline. This positive correlation persisted after 10–12 weeks of treatment; however, it ceased to be significant at the 24-week follow-up, even though the positive trend remained. This finding suggests that VDR expression may have a more pronounced influence on immune cells in cases of severe psoriasis, and this influence diminishes as the disease improves and immune status and responses normalize. On the other hand, despite varying levels of VDR expression over time, patients with psoriasis exhibited a positive response to the Etanercept treatment, as evidenced by a decrease in PASI scores [5]. This suggests that VDR expression on PBMCs might not be correlated with the severity of psoriasis.

In a study conducted on patients with chronic liver disease that investigated the presence of different expression levels of VDR proteins in PBMCs, it was found that the levels of CD3^+^VDR^+^ and CD14^+^VDR^+^ were significantly higher in hepatitis C virus-positive patients and patients with liver cirrhosis compared to healthy controls [14]. This aligns with our results showing higher VDR expression on CD14^+^ cells in patients with psoriasis compared to healthy controls, indicating that disease severity and inflammation might impact VDR expression. In the same study, they also found that VDR expression levels were associated with the clinical severity of liver disease; however, we did not observe a similar relationship in patients with psoriasis [14].

Vandikas et al. [5] found that psoriasis patients treated with Etanercept with sufficient levels of serum 25(OH)D at the start of TNFi improved on the Visual Analogue Scale (VAS) more rapidly than those with insufficient levels of serum 25(OH)D. A possible combination therapy of vitamin D and TNFi to secure vitamin D level sufficiency could help us to better understand the interplay between vitamin D and VDR expression in patients with psoriasis, as our findings suggest that the levels of serum vitamin D are likely to affect VDR expression, even though we could not find a statistically significant correlation between the two. Another study by Winter et al. [15] found that patients with inflammatory bowel disease (IBD) demonstrating normal vitamin D levels (the lower cut-off for normal vitamin D levels ranged from 9 to 33 ng/mL) at the initiation of TNFi treatment exhibited a 2.64-fold increased likelihood of achieving remission at three months compared to those with low vitamin D levels at treatment initiation. This finding suggests that vitamin D levels may influence the initial response to TNFi treatment, and that low levels of vitamin D may predispose patients to a reduced likelihood of remission.

A previous in vitro study on patients with rheumatoid arthritis observed that TNF blockade does not suppress the production of IL-17A and IL-22. However, a combination with 1,25(OH)_2_D could control human Th17 activity and synergistically inhibit synovial inflammation [16]. This possible synergistic effect between 1,25(OH)_2_D and anti-TNF-α is an interesting area of further research.

Given the fact that vitamin D can regulate the proliferation of keratinocytes, VDR impairment in epidermal skin could have a potential role in the pathogenesis of psoriasis, explaining why further research in this topic is necessary [2]. Chandra et al. [17] demonstrated that VDR is expressed in psoriatic skin. Moreover, they found a significant negative correlation between VDR expression in the skin and the PASI score. This contrasts with our findings, where no correlations were found between the PASI score and VDR expression on PBMCs. Elgarhy et al. found a significant increase in VDR expression at the sites of psoriasis lesions post-narrow-band ultraviolet B phototherapy (NB-UVB) compared to pretreatment lesional skin [18]. Prior to treatment, there was a significant decrease in VDR expression in psoriatic lesions compared to nonlesional skin. The same study found a negative correlation between the degree of VDR expression before treatment and the PASI score. On the other hand, Visconti et al. [19] observed reduced VDR expression in psoriatic skin, like the findings by Kim et al. [20], who also observed reduced VDR expression in psoriasis and perilesional skin compared to normal skin. Contradictory data on VDR expression suggests that further studies are required.

There is significant evidence suggesting that CD14 contributes to the pathogenesis of autoimmune conditions, which is also supported by its elevated levels in patients with vasculitis, systemic sclerosis, and psoriasis [21]. In the present study, there was a higher percentage of VDR expression on CD14^+^ monocytes compared to expression on CD3^+^ lymphocytes. This could indicate that the VDR might have a more pronounced influence on this subgroup of immune cells. Since CD14 plays a role in the pathogenesis of autoimmune diseases, further research is necessary [21]. In a flow cytometry analysis of VDR expression on PBMCs in patients with liver cirrhosis, hepatitis C virus positive patients, and healthy controls, median fluorescence intensity was higher for VDR expression on CD14^+^ cells compared to CD3^+^ cells [14].

Several previous studies, including a meta-analysis, have reported lower serum 25(OH)D levels in patients with psoriasis compared to controls, which contrasts with our findings [22]. However, while most studies report lower 25(OH)D levels in patients with psoriasis compared to controls, several studies have found no difference at all [23,24,25,26]. The varying results in previous research highlight the challenges of vitamin D research, particularly given the diverse causes of deficiency in individuals with psoriasis. Factors such as skin type, geographical region, dietary habits, age, comorbidities, and medications can significantly influence vitamin D levels. Additionally, differences in sun exposure and genetic factors further complicate our understanding of vitamin D deficiency in this population.

The main limitation of this study is the small number of participants. In the present study, we observed great individual variations in VDR expression (%) on both CD3^+^ and CD14^+^ cells among patients with psoriasis and in healthy controls, both at baseline and after 24 weeks. Given the small sample size, these variations in VDR expression could influence our results. A larger cohort of participants would be beneficial to the robustness of results. Additionally, the study was conducted throughout the entire calendar year, introducing seasonal variations that might impact our results [27]. Previous studies support the importance of VDR polymorphisms and their correlations with the severity of psoriasis [28]. In our study, we did not analyze VDR polymorphisms; however, all patients and controls included were from the same geographical region in West Sweden.

## 4. Materials and Methods

### 4.1. Study Population

Twenty patients with psoriasis were recruited from the outpatient clinic at the Department of Dermatology and Venereology at Sahlgrenska University Hospital, Gothenburg, Sweden, between 2013 and 2017. Fifteen (age- and sex-matched) individuals, without a history of psoriasis, other skin diseases, or other inflammatory disorders were included as controls.

Inclusion criteria for patients with psoriasis were age ≥18 years, moderate to severe plaque psoriasis (defined as: Psoriasis Area Severity Index (PASI) ≥10 and/or Dermatology Life Quality Index (DLQI) ≥10) where other systemic treatments were ineffective or considered inappropriate.

Exclusion criteria for both groups were: pregnancy or lactation, the presence of another ongoing severe chronic or systemic disease, treatment with oral steroids or other immunosuppressive/anti-inflammatory drugs, antibiotic treatment, and no previous TNFi exposure.

The study excluded participants with ongoing sunbed use, sunbed use or a sunny holiday in the past 4 weeks, and those using vitamin D-interactive medications (e.g., anticonvulsants), vitamin D supplements, or topical vitamin D analogues.

### 4.2. Study Design

Patients with psoriasis received treatment with Etanercept, administered subcutaneously at a dosage of 50 mg once a week for a duration of 24 weeks. Etanercept is a biologic fusion protein that functions as a soluble TNF inhibitor [29].

The psoriasis diagnosis was confirmed and skin type determined (according to Fitzpatrick) by an experienced dermatologist before treatment [30]. Patients with psoriasis were examined by a dermatologist at baseline, after 10–12 weeks, and after 24 weeks of treatment. Healthy controls were examined by a dermatologist at baseline. Disease severity was assessed by the PASI at each visit. The DLQI was used as a patient-reported outcome measure at each visit. At each visit, a questionnaire was completed, including medication, dietary supplements, medical history, sun habits, and other lifestyle variables that could affect inflammation and vitamin D status. Blood pressure, weight, height, and waist circumference were measured at every visit and body mass index (BMI) was calculated. Biochemical analyses were performed at every visit. According to the Endocrine Society, total serum levels of 25(OH)D ≥75 nmol/L were defined as sufficient, 50 to 74 nmol/L were defined as insufficient, and <50 nmol/L were considered deficient [31].

### 4.3. Flow Cytometry Detection of VDR and Biochemical Analyses of Vitamin D

Subpopulations of lymphocytes in blood were determined by flow cytometry.

Heparin blood was lysed using an ammonium chloride buffer (pH: 7.3) according to a standard procedure and washed in washing solution A (PBS with 3% (*v/v*) fetal bovine serum, 0.09% (*w*/*v*) sodium acid, and 0.5 mM EDTA, BD Biosciences, Mountain View, CA, USA (BD)). The cells were fixed and permeabilized using FixPerm (AH Diagnostics AB, Solna, Sweden (AH)) according to instructions by the manufacturer. After incubation in 4° C for 30 min, the cells were washed with FACSFlow followed by Perm buffer (both from BD). Subsequently, the following antibodies were added to the cells: fluorescein isothiocytanate-conjugated anti-CD3 (BD), APC-conjugated anti-CD14 (BD), and biotin-conjugated anti-VDR (VDR Ab-1 Clone 9A7g.E10.E4, Thermo Fisher Scie4ntific, Runcorn, Chesire, UK). The cells were incubated in 4 °C for 30 min and were then washed in Perm buffer. PE-conjugated streptavidin was added, and the cells were incubated in 4 °C for 30 min. After one wash in Perm buffer, the cells were suspended in washing solution A. Cell analysis was done on a flow cytometer (FACSCanto II, BD), which was controlled daily with CST (Cytometer Setup and Tracking)—beads in DIVA and with seven-color setup beads in FacsCanto Software (version number v.3.0.4894.41215), both from BD. In the staining of intracellular molecules, a lymphocyte gate was set manually according to location in a forward scatter versus side scatter diagram. Dot plots and quadrant statistics from the four-color analysis were generated by DIVA software (version number v.8.0.1) (BD). The results for each subpopulation were expressed as the percentage of monocytes and T lymphocytes, respectively, and as the number of cells × 10^9^/L. To analyze the total 25(OH)D [25(OH)D2 and 25(OH)D3] an electrochemiluminescence immunoassay (ECLIA) on a Cobas 8000 Roche instrument (Roche Diagnostics Scandinavia AB, Tokyo, Japan) using an Elecsys vitamin D Total II assay was used. A two-step immunosorbent assay (enzyme-linked immunoassay; ELISA) performed on a commercial kit (Future Diagnostics B.V., Wijchen, The Netherlands) was used to measure the free 25(OH)D concentration.

### 4.4. Statistics

All data were analyzed using R version 3.5.3 (The R Foundation for Statistical Computing, Vienna, Austria). Spearman’s correlation test was used to test for univariate correlations. Wilcoxon rank sum test was used for comparisons between groups. Spearman’s correlation test, stratified with respect to patient, was used for comparing changes in time within each group. All tests were two-sided and *p* < 0.05 was considered statistically significant.

### 4.5. Ethical Considerations

The study was approved by the Ethics Committee at the University of Gothenburg on 22 May 2012 (approval number: 089-12). The Declaration of Helsinki’s protocols were followed.

## 5. Conclusions

Patients with moderate to severe psoriasis had higher VDR expression on CD14^+^ cells compared to healthy controls. Treatment with Etanercept for 24 weeks did not alter VDR expression on CD3^+^ and CD14^+^ cells in this cohort of patients with psoriasis with sufficient serum vitamin D levels at the treatment’s start, implying that VDR expression might not be related to disease severity or the grade of inflammation. Moreover, with an increase in total and free 25(OH)D levels, VDR expression on CD14^+^ cells also increased in healthy controls over time, strengthening the hypothesis that 25(OH)D, rather than disease severity, influences VDR expression. However, it is important to note that we did not find a statistically significant correlation between total or free 25(OH)D levels and VDR expression in this study. On the other hand, we found a positive correlation between VDR expression on CD3^+^ lymphocytes and CD14^+^ monocytes in patients with psoriasis before the treatment’s start, which ceased to be significant after 24 weeks of the Etanercept treatment. This finding could indicate that VDR is more involved in the interplay between these cells during severe inflammation. In contrast to other studies analyzing VDR in the skin, we did not find any correlations between the PASI and VDR expression on PBMCs in this group of patients with psoriasis.

## Figures and Tables

**Table 1:** Baseline demographic data for patients with psoriasis and healthy controls. Mean values ± standard deviations (SD) and *p*-values for comparison between the two groups are presented.

	Patients with Psoriasis		Healthy Controls		
	*n* = 20		*n* = 15		
	Mean (SD)	*n*	Mean (SD)	*n*	*p*-Value

Age (years)					
Men	48 (12)	13	50 (9)	10	0.26 ^a^
Women	56 (15)	7	52 (10)	5	0.64 ^a^
All	51 (13)	20	51 (9)	15	0.63 ^a^
Duration of psoriasis (years)	28 (12)	19			
Skin type	*n* (%)		*n* (%)		
II	7 (35%)		2 (13%)		0.37 ^b^
III	12 (60%)		12 (80%)		
IV	1 (5%)		1 (7%)		
	*n* (%)		*n* (%)		
Self-reported arthropathy	15 (75%)		0 (0%)		
Current smokers	8 (40%)		1 (7%)		
Antidyslipidemic use	4 (20%)		1 (7%)		
Antihypertensive use	3 (15%)		1 (7%)		
Antidiabetic use	1 (5%)		0 (0%)		
Antidepressant use	3 (15%)		0 (0%)		
Painkiller use	4 (20%)		0 (0%)		
Hypothyroidism medication	0 (0%)		2 (13%)		
Hormonal contraception	0 (0%)		0 (0%)		
Aspirin	1 (5%)		0 (0%)		
Obesity (Body Mass Index ≥ 30 kg/m^2^)	6 (30%)		5 (33%)		

^a^ Wilcoxon rank sum test. ^b^ Fisher’s exact test.

**Table 2:** Expression of the vitamin D receptor (VDR) on CD3^+^ lymphocytes and CD14^+^ monocytes, Psoriasis Area Severity Index (PASI), and vitamin D metabolites in patients with psoriasis before (baseline) and after (24 weeks) treatment with Etanercept, and in healthy, untreated controls at the same time points. Mean values ± standard deviations (SD) are presented.

		Patients with Psoriasis			Healthy Controls			
		*n* = 20			*n* = 15			
		Mean (SD)	*n*	*p*-Value for Trend Over Time ^a^	Mean (SD)	*n*	*p*-Value for Trend Over Time ^a^	*p*-Value; Difference at Baseline between Groups ^b^
CD3^+^ VDR (%)								
	Baseline	6.5 (8.7)	20	0.99	5.3 (6.9)	14	0.80	0.83
	24 weeks	9.5 (20)	17		6.1 (8.1)	12		
CD14^+^ VDR (%)								
	Baseline	54 (32)	20	0.96	28 (22)	14	0.035 *	0.015 *
	24 weeks	52 (35)	17		44 (31)	12		
PASI score								
	Baseline	13 (5)	20	<0.0001 ***				
	24 weeks	3 (3)	17					
25(OH)D (nmol/L)								
	Baseline	74 (31)	20	0.34	53 (19)	14	0.029 *	0.018 *
	24 weeks	76 (23)	17		73 (20)	12		
Free25(OH)D (pmol/L)								
	Baseline	11.8 (4.0)	20	0.84	8.0 (2.6)	15	0.006 **	0.001 **
	24 weeks	11.7 (2.8)	17		10.2 (2.9)	12		
1,25(OH)2D (pmol/L)								
	Baseline	103 (34)	20	0.68	91 (36)	15	0.088	0.33
	24 weeks	96 (20)	17		112 (38)	12		

^a^ Spearman’s test, stratified with respect to patient. ^b^ Wilcoxon rank sum test. * *p* < 0.05, ** *p* < 0.01, *** *p* < 0.001.

## Data Availability

Upon reasonable request, data supporting the findings of the study will be available from the corresponding author.
